# N-linoleyltyrosine ameliorates high-fat diet-induced obesity in C57BL/6 mice *via* cannabinoid receptor regulation

**DOI:** 10.3389/fendo.2022.938527

**Published:** 2022-08-30

**Authors:** Zheng-yu Yang, Yi-ying Wu, Yi Zhou, Yun-qi Yang, Jia-hui Zhang, Tao He, Sha Liu

**Affiliations:** ^1^ Department of Pharmacy, Sichuan Province College Key Laboratory, Chengdu Medical College, Chengdu, China; ^2^ Department of Thoracic Surgery, The Second Affiliated Hospital of Chengdu Medical College, China National Nuclear Corporation 416 Hospital, Chengdu, China; ^3^ Research and Development Center, Sichuan Yuanda Shuyang Pharmaceutical Co., Ltd, Chengdu, China

**Keywords:** N-linoleyltyrosine, endocannabinoid, diet-induced obesity, cannabinoid receptor, glucose and lipid metabolism

## Abstract

**Objectives:**

N-linoleyltyrosine (NITyr) showed mild effects in preclinical studies. The research discussed the effect of NITyr on a high-fat diet (HFD) induced obese (DIO) mice, and preliminarily explored its mechanism.

**Methods:**

The DIO mice were established by feeding an HFD for 12 weeks and subsequently administrated orally with NITyr (30, 60 and 100 mg/kg) for four weeks. The indexes of serum and liver samples were determined by ELISA kit. The pathological status of adipose and liver were detected by HE staining. The factors related to energy and lipid metabolism were measured *via* western blot.

**Results:**

NITyr at 60 and 100 mg/kg/day suppressed the weight gain without affecting water and food intake. Accordingly, NITyr reduced adipose weight and the area of individual adipocytes and increased the number of adipocytes. Moreover, NITyr didn’t affect the appetite-related indexes such as ghrelin, peptide YY and brain-derived neurotrophic factor. Besides, NITyr didn’t affect other organ coefficients except for the liver. Correspondingly, NITyr reduced alanine aminotransferase and aspartate aminotransferase levels, yet didn’t influence IL-1β and TNF-α levels, and the liver injury. The levels of triacylglycerol (TG), total cholesterol (TC), glucose, insulin, adiponectin and leptin in serum were assessed to evaluate the effect of NITyr on glucose and lipid metabolism. NITyr decreased the levels of TG, TC and glucose, and didn’t affect insulin, adiponectin and leptin levels. Meanwhile, NITyr up-regulated p-AMPK and the cannabinoid receptor 2 (CB_2_) expressions, and down-regulated PPAR, FAS and cannabinoid receptor 1 (CB_1_) expressions.

Overall, NITyr suppressed lipid accumulation *via* improving lipid and glucose metabolism involving CB_1_ and CB_2_ receptors.

## Introduction

Obesity is defined as an imbalance between caloric intake and energy consumption (1). Obese people are often accompanied by abnormal blood sugar, blood lipids, blood pressure, and insulin levels, and with prone to diabetes, hypertension, cardiovascular and cerebrovascular diseases (2).

The World Health Organization estimated that two out of five adults worldwide will be obese by 2030. Within several decades, obesity has become a global problem (3). Except for obesity caused by genetic and pathological factors, most obesity is diet-induced obesity (DIO) (4). The effect of exercise and diet intervention in losing weight is not satisfactory enough, and long-term intervention with drugs is required (5, 6). Medications used for obesity treatment such as cannabinoid receptor 1 (CB_1_) antagonist Rimonabant with side effects of either depression or gastrointestinal reactions, respectively (7), resulting in low patient compliance. Since obesity is closely associated with the disorder of glucose and lipid metabolism, hormone disturbance and low-grade inflammation (8, 9), therefore, a compound comprehensively intervening in the above pathological pathways, while not affecting drinking and appetite, has advantages over traditional combinations in promoting weight loss and safety concerns.

N-linoleoyltyrosine (NITyr), an endocannabinoid analog, exerts neuroprotective effects in APP/PS1 transgenic mice, protects against transient cerebral ischemia in gerbils, and protects PC12 cells against oxidative damage *via* mediating cannabinoid receptors (CB_1_ and CB_2_) as a neuroprotective agent *in vitro* (10–12). CB_1_ and CB_2_ are potential therapeutic targets for obesity (13–15). CB_1_ is highly expressed in the central nervous system, as well as adipose, muscle, adrenal gland, liver, gastrointestinal tract and other tissues (16). CB_1_ activation improves glucose uptake and increases peroxisome proliferator-activated receptor gamma (PPAR-γ) and lipoprotein lipase expressions, which promote adipocyte proliferation and increase the size and quantity of triglyceride in adipocytes of diet-induce obese mice (17). Additionally, CB_1_ activation decreases adiponectin expression and increases leptin expression in mouse white adipose tissue (18). Moreover, CB_1_ activation causes an expansion of the adipose tissue in the liver (19). CB_2_ is mainly distributed in brain regions related to appetite, and peripheral regions, metabolically active, such as liver, adipose, skeletal muscle, islets, etc. (20). Meanwhile, CB_2_ activation improves insulin sensitivity, energy homeostasis and inflammation (21). And 60 mg/kg/day NITyr promotes weight loss (data unpublished). Importantly, NITyr improves the learning and memory ability of mice through CB_1_ and CB_2_ receptors, but not anxiety and depression in Alzheimer’s disease. It should be noted that NITyr produces positive effects on metabolic pathologies. Therefore, all these characters mentioned above highlight the need for further research for NITyr on obesity.

In the present study, the anti-obese effect and possible mechanisms of NITyr were confirmed. Firstly, a DIO model was established, and the basic information of mice such as drinking, appetite and body weight were recorded. Next, the glucose and lipid metabolism related factors were measured. Furthermore, whether the effect of NITyr was associated with CB_1_ and CB_2_ was discussed.

## Materials and methods

### Materials

NITyr was independently synthesized in our laboratory according to the literature (12), Orlistat (MACKLIN, purity: 98%, CAS: 96829-58-2), Poloxamer 188 (Solarbio, CAS: 9003-11-6), 45% kcal high-fat diet (MD12032, Medicine, Jiangsu, China; protein 24%, fat 24% and carbohydrate 41%), RIPA lysis Buffer (Strong) (Cwbio, CW2333), SDS-PAGE Loading Buffer (Cwcio, CW0027S, 5 ×), Protease inhibitor cocktail (Cwbio, CW22005, 100 ×), Phosphatase inhibitor cocktail (Cwbio, CW2383S, 100 ×), CNR1 Ab - DF4918 (Source: Rabbit, Cat. #: DF4918, Affinity Biosciences), CNR2 Ab - DF8646 (Source: Rabbit, Cat. #: DF8646, Affinity Biosciences), GAPDH (Source: Rabbit, Cat. #: AF7021, Affinity Biosciences), FAS Ab - AF5342 (Source: Rabbit, Cat. #: AF492, Affinity Biosciences), PPAR gamma Ab - AF6284 (Source: Rabbit, Cat. #: AF6284, Affinity Biosciences), Phospho-AMPK alpha (Thr172) Antibody (Source: Rabbit, Cat. #: CY6027, Abways Technology), Goat Anti-Rabbit lgG (H + L) HRP - S0001 (Source: Goat, Cat.#: S0001, Affinity Biosciences), mouse insulin enzyme-linked reaction kit (MM-0579M, Meimian, Jiangsu), mouse adiponectin enzyme-linked reaction kit (MM-0547M, Meimian, Jiangsu), mouse leptin enzyme-linked reaction kit (MM-0622M, Meimian, Jiangsu), mouse ghrelin enzyme-linked reaction kit (MM-0621M, Meimian, Jiangsu), mouse peptide YY (PYY) enzyme-linked reaction kit (MM-0649M, Meimian, Jiangsu), mouse brain-derived neurotrophic factor (BDNF) enzyme-linked reaction kit (MM-0204M, Meimian, Jiangsu), mouse alanine aminotransferase (ALT) enzyme-linked reaction kit (MM-44625M, Meimian, Jiangsu), mouse aspartate aminotransferase (AST) enzyme-linked reaction kit (MM-4415M, Meimian, Jiangsu), mouse tumor necrosis factor ɑ (TNF-ɑ) enzyme-linked reaction kit (MM-0132M, Meimian, Jiangsu), mouse interleukin-1β (IL-1β) enzyme-linked reaction kit (MM-0040M, Meimian, Jiangsu), mouse triacylglycerol (TG) enzyme-linked reaction kit (A110-1-1, Jiancheng, Nanjing), mouse total cholesterol (TC) enzyme-linked reaction kit (A111-1-1, Jiancheng, Nanjing), mouse glucose enzyme-linked reaction kit (A154-1-1, Jiancheng, Nanjing).

### Animals and diets

The operations were approved by the Animal Experiment Ethics Committee of Chengdu Medical College. Seventy-two male 3-week-old C57BL/6 mice (Chengdu DaShuo, Sichuan, China) weighing ~11.8 g were fed (6 mice per cage) in an environment with 23 ± 1°C and 60 ± 5% humidity. The mice were allowed free access to food and water. After being adapted to a 12% kcal fat standard diet (MD12031, Medicine, Jiangsu, China; protein 19.2%, fat 4.3% and carbohydrate 67.3%) for one week, the mice were treated with NITyr and Orlistat. The mice were randomly divided into the control group (12 mice, fed 12% kcal fat standard diet) and the high-fat group (60 mice, fed 45% kcal high-fat diet). The body weights, food and water intake (Formula (1) (2),) were recorded weekly for 11 weeks. Then the mice with no significance in body weight between the high-fat group and the control group were removed (*P* > 0.05).

Daily food intake = (W_i_ - W_f_) ÷ N ÷ D, g/mouse/day (1)

Daily water intake = (V_i_ - V_f_) ÷ N ÷ D, mL/mouse/day (2)

In the formula, W_i:_ initial mass of feed; W_f_: final mass of feed; V_i_: initial water supply; V_f_: final water; N: number of mice per cage; D: days.

### Drugs treatment and tissue collection

Drug treatment began at the 13^th^ week and lasted for four weeks, and the feeding of each group followed the above method. The body weights, food and water intake (Formula (1) (2),) were recorded weekly. NITyr and Orlistat were suspended in 0.05 g/mL Poloxamer 188 aqueous solution, and then administered orally at 0.1 mL/10 g per day. The control group (mice served a standard diet) and the DIO1-DIO5 group (mice served a fat diet). The experiment group was as follows: the control group (the normal mice treated with Poloxamer 188 aqueous solution), The DIO group (the obesity mice treated with Poloxamer 188 aqueous solution), the 30 NITyr group (the obesity mice treated with 30 mg/kg NITyr), the 60 NITyr group (the obesity mice treated with 60 mg/kg NITyr), the 100 NITyr group (the obesity mice treated with 100 mg/kg NITyr), the Orlistat group (the obesity mice treated with 100 mg/kg Orlistat).

After drug intervention, mice were weighed and euthanized by cervical dislocation. Serum was prepared by keeping the blood at room temperature for 20 min until coagulation, and then centrifuged (4°C, 3,000 g for 10 min) and stored at - 80°C. The heart, spleen, liver, lung, kidney, brain and adipose tissues were removed, rinsed with phosphate-buffered saline (PBS: 135 mM NaCl, 2.7 mM KCl, 1.5 mM KH_2_PO_4_, and 8 mM K_2_HPO_4_, pH 7.2) at 4°C and weighted, then stored in - 80°C. The organ coefficient was calculated as Formula (3).

Organ coefficient = (organ weight/body weight) × 100, g/100 g (3).

### Biochemical analysis of serum and liver

TG, TC, glucose, insulin, leptin, ghrelin, PYY and BDNF levels in serum and ALT, AST, TNF-α and IL-1β levels in the liver were determined respectively using corresponding enzyme kits according to the manufacturer’s instructions.

### Histological analysis of liver and adipose tissue

The liver and adipose tissue were fixed in 4% paraformaldehyde solution (BL539A, BioSharp, Shanghai, China) for 48h, and subsequently embedded in paraffin. The embedded tissue was cut into slices with five μm-thick sections, subsequently stained with hematoxylin and eosin (H&E), and finally, the cellular structure and lipid accumulation of liver and adipocytes were observed under observed using a light microscope (BA210Digital.) under magnification 400 ×.

### Western blot

For protein extraction, brain tissues were chopped and weighed, and then its homogenate was lysed with RIPA solution for 30 min (tissue: RIPA = 0.1 g: 500 µL). Meanwhile, the protease inhibitors and phosphatase inhibitors were added to the above solutions. The operations were performed on ice. The lysates were centrifuged (4°C, 3,000 g, 10 min) and their supernatants were collected. The western blot method was consistent with the literature (16) and the primary antibody was changed (CNR1 Ab - DF4918, CNR2 Ab - DF8646).

### Statistical analysis

The data were represented by mean ± standard deviation (SD) and were analyzed by SPSS software. The statistical method applied in the study was one-way ANOVA, followed by the turkey test. A significant difference was obtained when *P* < 0.05.

## Results

### Establishment of the diet-induced obesity

**Figure 1:**
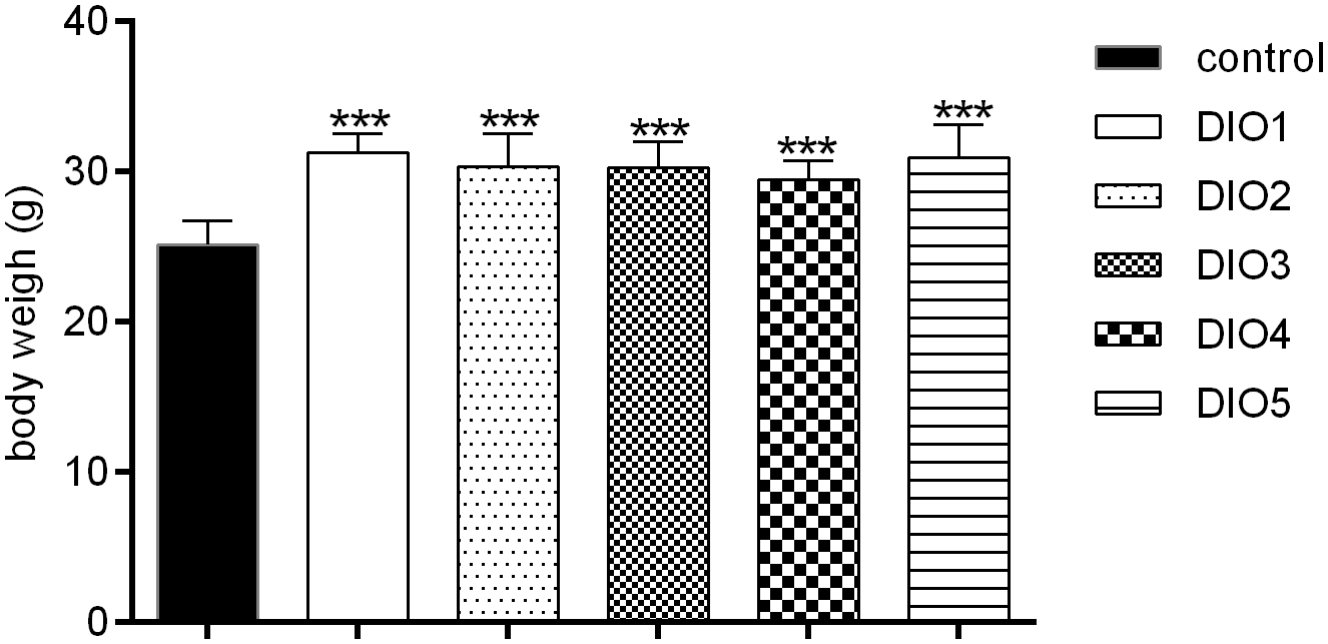
Body weight under 12-week HFD dietary intervention. Control was given a 12% kcal normal fat diet. DIO1-5 were given a 45% kcal high-fat diet. All values were expressed as means ± SD (n = 8). ^***^ *P* < 0.001, as compared with the control group.

The weight of mice was measured at the end of DIO establishment (**Figure 1**). Compared to the control group, the body weights of DIO mice was significantly increased (17-24%, *P* < 0.001) and no significance in body weights was observed among the DIO1-DIO5 group, indicating that the high-fat food (HFD) did induce obesity.

### Body weight during 4-week drug administration

**Figure 2:**
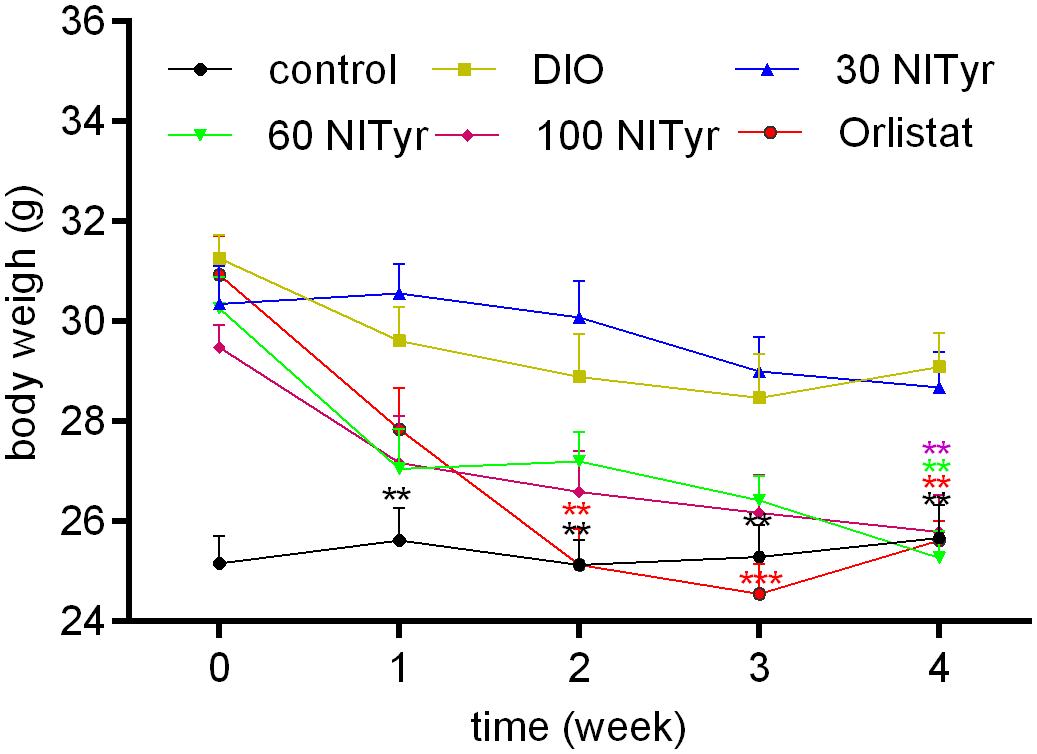
Figure 2 Body weight during 4-week drug intervention period. NITyr and Orlistat promoted weight loss of mice. Control or DIO was treated with Poloxamer 188 aqueous solution. 30 NITyr, 60 NITyr, 100 NITyr and Orlistat were treated with 30 mg/kg NITyr, 60 mg/kg NITyr, 100 mg/kg NITyr, 100 mg/kg Orlistat, respectively. All values were expressed as means ± SD (n = 8). ^**^ *P* < 0.01, ^***^ *P* < 0.001, as compared with the DIO group.

When the DIO mice were given Orlistat for two weeks, a significant decrease (*P* < 0.01) was observed in the Orlistat group as compared with the DIO group (**Figure 2**), until the DIO mice were treated with NITyr for four weeks, the body weights in the NITyr group decreased compared with the DIO group (all *P* < 0.01).

### Effect of NITyr on appetite

**Figure 3:**
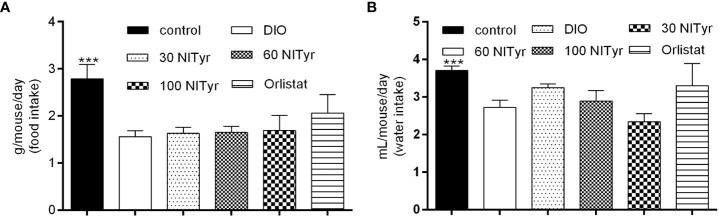
**(A)** Average food intake during 4-week drug intervention. **(B)** Average water intake during 4-week drug intervention. Control or DIO was treated with Poloxamer 188 aqueous solution. 30 NITyr, 60 NITyr, 100 NITyr and Orlistat were intervened with 30 mg/kg NITyr, 60 mg/kg NITyr, 100 mg/kg NITyr and 100 mg/kg Orlistat, respectively. All values were expressed as means ± SD (n = 4). ^***^ *P* < 0.001, as compared with the DIO group.

As shown in **Figure 3**, compared with the control group, the food intake and water intake in the DIO group were reduced (*P* < 0.001), and the treatment group didn’t attenuate the above phenomena. Meanwhile, the factors related to appetite were detected (**Table 1**). The levels of peptide YY (PYY), a feeding inhibitor, and ghrelin, a feeding stimulator, were measured. The levels of ghrelin and PYY increased in the DIO group compared with that of the control group, NITyr didn’t affect the above factors. Besides, compared with the control group, the levels of brain-derived neurotrophic factor (BDNF) were decreased in the DIO group, and no significance in the BDNF level was investigated after NITyr intervention.

**Table 1:** Analysis of several appetite indexes in serum of mice after drug administration for 4 weeks.

Value	control	DIO	30NITyr	60 NITyr	100 NITyr	Orlistat
Ghrelin (ng/L)	133.58 ± 34.36*	234.65 ± 55.62	201.46 ± 28.44	224.56 ± 54.96	262.62 ± 35.65	191.20 ± 47.82
PYY (pg/mL)	62.69 ± 10.21*	106.26 ± 29.57	71.19 ± 15.13	95.51 ± 20.05	70.82 ± 13.92	76.76 ± 13.85
BDNF (ng/L)	644.05 ± 127.62*	408.97 ± 135.62	296.22 ± 132.35	336.76 ± 60.88	321.91 ± 105.15	304.89 ± 129.81

All values were expressed as means ± SD (n = 3). ^*^P < 0.05, as compared with the DIO group.

### Effect of NITyr on organ coefficient in mice

A significant difference (*P* < 0.05, *P* < 0.001) in the organ coefficient of liver and adipose was observed in the DIO group compared to the control group (**Table 2**), and NITyr decreased the above factors. Besides, no significance was observed in the organ coefficient conclude heart, spleen, lung, kidney and brain among all groups.

**Table 2:** Effect of NITyr on organ coefficient in mice.

Value	control	DIO	30 NITyr	60 NITyr	100 NITyr	Orlistat
Heart	0.46 ± 0.04	0.50 ± 0.10	0.38 ± 0.06	0.45 ± 0.73	0.46 ± 0.09	0.60 ± 0.13
Liver	3.87 ± 0.14*	3.06 ± 0.45	2.76 ± 0.74	4.04 ± 0.41**	3.91± 0.27**	4.43 ± 0.28***
Spleen	0.29 ± 0.08	0.35 ± 0.12	0.29 ± 0.04	0.35 ± 0.05	0.34 ± 0.09	0.44 ± 0.13
Lung	0.64 ± 0.14	0.58 ± 0.11	0.60 ± 0.08	0.57 ± 0.04	0.60 ± 0.11	0.66 ± 0.07
Kidney	1.10 ± 0.04	1.37 ± 0.25	1.14 ± 0.24	1.35 ± 0.06	1.30 ± 0.12	1.10 ± 0.26
Brain	1.38 ± 0.24	1.33 ± 0.08	1.04 ± 0.17	1.26 ± 0.16	1.36 ± 0.24	1.34 ± 0.20
Adipose	1.11 ± 0.32***	5.07 ± 1.52	3.57 ± 1.03	3.28 ± 0.89*	3.15 ± 1.42*	2.30 ± 0.89**

All values were expressed as means ± SD (n = 8). ^*^P < 0.05, ^**^P < 0.01 and ^***^P < 0.001, as compared with the DIO group.

### Effect of NITyr on adipocyte

**Figure 4:**
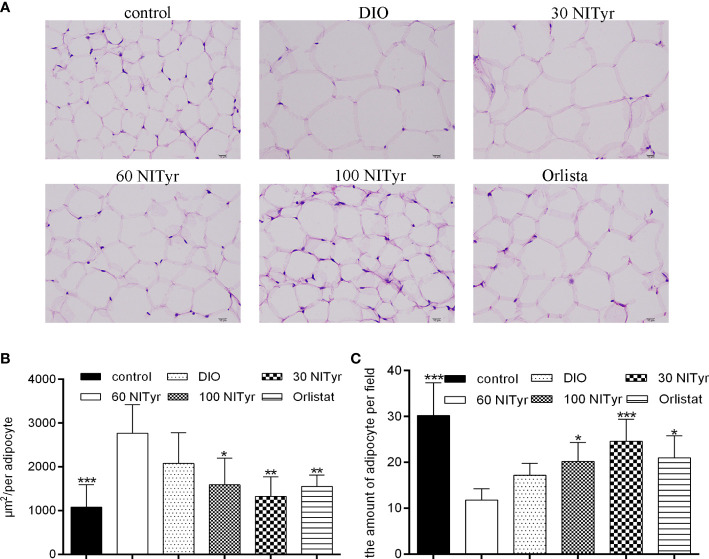
**(A)** The adipose tissue of mice stained by hematoxylin and eosin (H&E). scar bar = 10 µm. **(B)** The area of adipocytes in each group under the same field. **(C)** the number of adipocytes in each group of mice under the same field. The control or the DIO group was treated with Poloxamer 188 aqueous solution. 30 NITyr, 60 NITyr, 100 NITyr and Orlistat were treated with 30 mg/kg NITyr, 60 mg/kg NITyr, 100 mg/kg NITyr and 100 mg/kg Orlistat, respectively. All values were expressed as means ± SD. (n = 4). ^*^ *P* < 0.05, ^**^ *P* < 0.01, ^***^ *P* < 0.001, as compared with the DIO group.

As shown in **Figure 4**, compared with the control group, the adipocyte area in the DIO group increased and the number of adipocytes decreased in each field (*P* < 0.001, *P* < 0.001). The image of adipocytes showed an expansion in the DIO group compared with the control group, suggesting HFD-induced adipose tissue hypertrophy. NITyr attenuated the above phenomena, indicating that NITyr inhibited adipocyte hypertrophy.

### Effect of NITyr on liver

**Figure 5:**
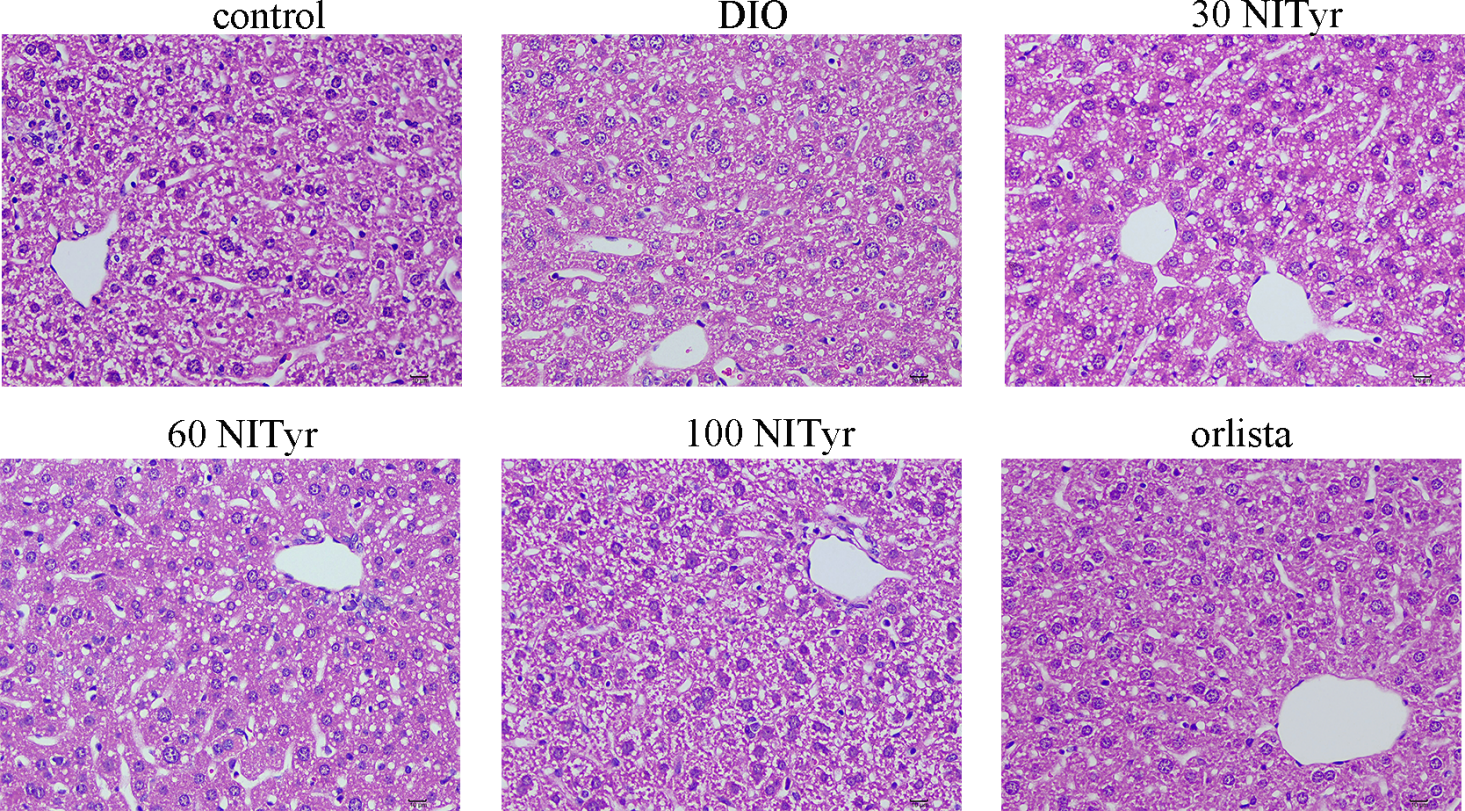
The liver tissue of mice stained by the H&E method. scar bar = 10 µm. The control or DIO group was treated with Poloxamer 188 aqueous solution. 30 NITyr, 60 NITyr, 100 NITyr and Orlistat were treated with 30 mg/kg NITyr, 60 mg/kg NITyr, 100 mg/kg NITyr, 100 mg/kg Orlistat, respectively.

The levels of ALT, AST, IL-1β and TNF-ɑ correlated with liver injury and inflammation were tested (**Table 3** **)**. All the above indicators increased significantly (*P* < 0.01, *P* < 0.05, *P* < 0.05, *P* < 0.05, respectively) in DIO group compared with the control group. NITyr treatment (100 mg/kg) weakened ALT and AST levels, while didn’t affect IL-1β and TNF-ɑ levels. The H&E staining of the liver showed that the hepatic injury didn’t exist among all groups (**Figure 5** **)**.

**Table 3:** Analysis of liver injury and inflammation indexes in serum in mice after 4-week administration of drugs.

Value	control	DIO	30 NITyr	60 NITyr	100 NITyr	Orlistat
ALT (ng/L)	28.92 ± 5.97**	53.81 ± 15.40	38.06 ± 9.36	41.11 ± 7.45	35.21 ± 9.38*	36.92 ± 9.73
AST (ng/L)	31.84 ± 11.79*	55.50 ± 14.46	50.49 ± 16.09	34.30 ± 11.67	33.70 ± 14.00*	38.45 ± 13.04
IL-1β (ng/L)	199.84 ± 11.79*	372.33 ± 57.54	251.87 ± 43.85	350.29 ± 106.20	387.87 ± 115.97	446.74 ± 108.79
TNF-α (ng/L)	18.71 ± 6.42*	33.78 ± 8.24	29.45 ± 6.92	37.63 ± 6.58	34.68 ± 9.87	41.81 ± 5.85

All values were expressed as means ± SD (n = 4). ^*^P < 0.05, ^**^P < 0.01, as compared with the DIO group.

### Effect of NITyr on glucose and lipid metabolism

TG, TC, glucose, insulin, adiponectin and leptin were detected to investigate the effects of NITyr on lipid and carbohydrate metabolism (**Table 4** **)**. Compared with the control group, the levels of TG, TC, glucose and insulin in the DIO group were significantly increased (*P* < 0.001, *P* < 0.01, *P* < 0.001, and *P* < 0.01, respectively). In contrast, the levels of adiponectin and leptin were descended (*P* < 0.05, *P* < 0.05, respectively), indicating that HFD led to an imbalance of lipid and carbohydrate metabolism. NITyr decreased TG, TC and glucose levels in DIO mice (*P* < 0.05, *P* < 0.05, *P* < 0.01, respectively), yet didn’t affect the level of insulin, adiponectin and leptin.

**Table 4:** Analysis of glucose and metabolism indexes in serum in mice after 4-week administration of drugs.

Value	control	DIO	30 NITyr	60 NITyr	100 NITyr	Orlistat
TG (mM)	1.49 ± 0.06***	1.87 ± 0.14	1.71 ± 0.21	1.66 ± 0.13*	1.63 ± 0.10*	1.53 ± 0.15**
TC (mM)	2.36 ± 0.62**	3.94 ± 0.83	3.32 ± 0.96	2.66 ± 0.34*	2.75 ± 0.22*	2.47 ± 0.88*
Glucose (mM)	4.51 ± 0.71***	8.81 ± 1.29	6.55 ± 1.67	5.45 ± 1.58**	5.19 ± 0.90**	6.46 ± 1.69*
Insulin(mIU/L)	0.42 ± 0.07**	0.63 ± 0.09	0.54 ± 0.09	0.56 ± 0.13	0.51 ± 0.06	0.47 ± 0.06
Adiponectin (µg/L)	139.49 ± 13.16*	104.73 ± 16.2	131.61 ± 16.23	131.43 ± 17.99	132.36 ± 18.62	140.79 ± 19.84*
Leptin (pg/L)	556.75 ± 106.33*	410.25 ± 38.49	422.56 ± 43.73	545.68 ± 83.70	521.42 ± 73.89	556.83 ± 69.16*

All values were expressed as means ± SD (n = 8). ^*^P < 0.05, ^**^P < 0.01, ^***^P < 0.001, as compared with the DIO group.

### Effect of NITyr on the expression of key factors in adipogenesis

**Figure 6:**
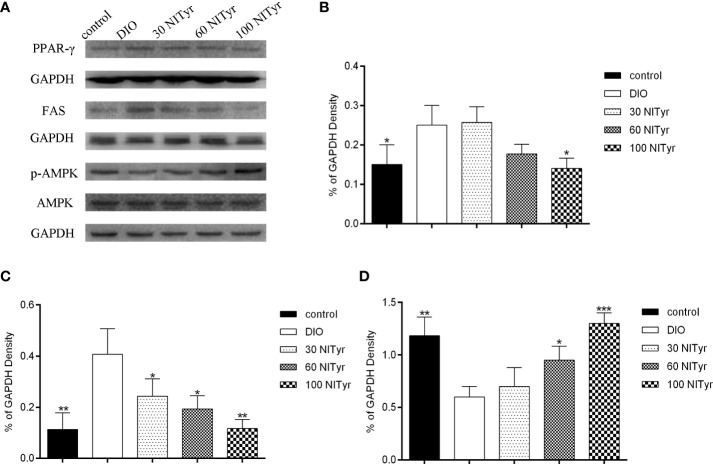
Effect of NITyr on the expression of key factors in adipogenesis. **(A)** Western blot analysis of PPAR-γ, FAS and p-AMPK. **(B)** PPAR-γ expressions were normalized that of GAPDH. **(C)** FAS expressions were normalized that of GAPDH. **(D)** p-AMPK expressions were normalized that of AMPK. The control or DIO group was treated with Poloxamer 188 aqueous solution. 30 NITyr, 60 NITyr and 100 NITyr were treated with 30 mg/kg NITyr, 60 mg/kg NITyr, 100 mg/kg NITyr, respectively. All values were expressed as means ± SD. (n = 3). ^*^ *P* < 0.05, ^**^ *P* < 0.01, ^***^ *P* < 0.001, as compared with the DIO group.

As shown in **Figure 6**, NITyr treatment noticeably suppressed the elevated expression of PPAR (*P* < 0.05) and FAS (*P* < 0.05, *P* < 0.05, and *P* < 0.01, respectively) in DIO group. The p-AMPK expression was significantly decreased in the DIO group compared with the control group but was increased by NITyr treatment (*P* < 0.05, and *P* < 0.01, respectively).

### Effect of NITyr on CB_1_ and CB_2_ protein levels in DIO mice

**Figure 7:**
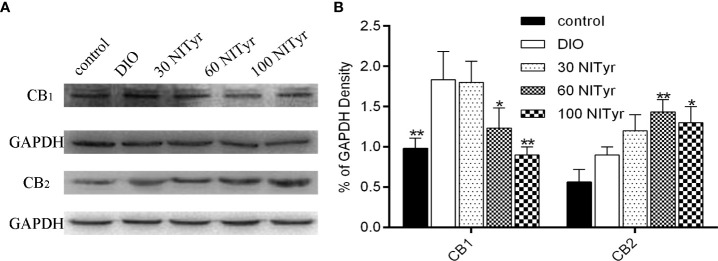
Effect of NITyr on the expression of CB_1_ and CB_2_ in brain. **(A)** Western blot analysis of CB_1_ and CB_2_. **(B)** CB_1_ and CB_2_ expressions were normalized that of GAPDH. The control or DIO group was treated with Poloxamer 188 aqueous solution. 30 NITyr, 60 NITyr and 100 NITyr were treated with 30 mg/kg NITyr, 60 mg/kg NITyr, 100 mg/kg NITyr, respectively. All values were expressed as means ± SD. (n = 3). ^*^ *P* < 0.05, ^**^ *P* < 0.01, as compared with the DIO group.

Compared with the control group, the CB_1_ expressions were upregulated in the DIO group (*P* < 0.01) and no significance was observed in CB_2_ expressions (**Figure 7**). Besides, NITyr treatment downregulated CB_1_ expressions (*P* < 0.05, *P* < 0.01) while upregulated CB_2_ expressions (*P* < 0.01, *P* < 0.05).

**Figure 8:**
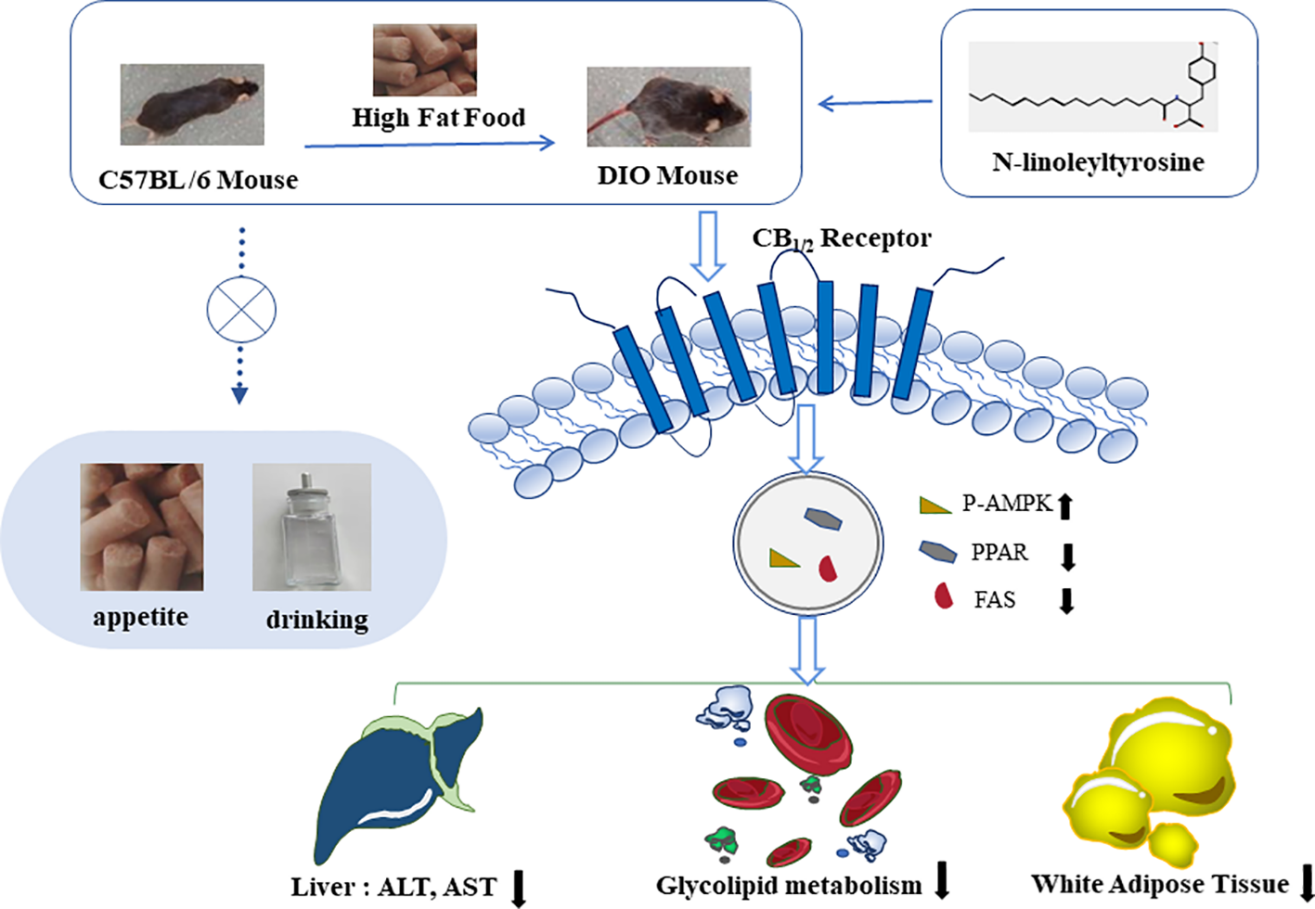
N-linoleyltyrosine ameliorates high-fat diet-induced obesity in C57BL/6 mice via cannabinoid receptor regulation, involving in regulating lipid and glucose metabolism, but not appetite.

## Discussion

Obesity is a chronic metabolic disorder caused by genetic or environmental factors (22). When energy intake is greater than energy consumption, the body will cause fat accumulation, resulting in obesity. Establishing an obesity model suitable for the experiment is a prerequisite for obesity study. Nutritional obesity without definite etiology was caused by energy intake exceeding consumption (23). The nutritional obesity model induced by HFD is widely used, similar to human obesity (24, 25). Therefore, we use the commercially available high-fat feed to establish the obesity model, which is stable and straightforward. As C57BL/6 mice are sensitive to an HFD, they are constantly applied to obesity (26). Orlistat, a weight-loss drug, is associated with gastrointestinal reactions, but it exerted benign efficacy and high safety compared with the other weight-loss drugs such as naltrexone and bupropion (27). Hence, it was selected as the positive drug in the experiment. The DIO mice were induced by continuous feeding with HFD for 12 weeks according to the method of Tang (28). Because some mice own obesity genes and love to grab food, free-feeding leads to individual differences in the weight of mice. Thus, in the study, mice with no significance compared with the control group were excluded. Meanwhile, mice with no difference in weight between the high-fat groups were retained as the model mice for subsequent experiments.

Body weight directly reflects the weight loss activities of drugs. In the first week of drug intervention, the weight loss of mice decreased sharply, but it became gentle in the later stage of drug intervention. We speculated that the intragastrical administration reduced the food intake of mice, resulting in significant weight loss. As the mice gradually adapted to the intragastrical continuous administration, the weight loss became gentle. Meanwhile, the mice treated with Orlistat showed side effects such as loose stool and malaise, while mice treated with NITyr didn’t show the above reaction. Thus, NITyr owns a good safety. On account of the efficacy of NITyr on weight loss, the effect of NITyr on organ coefficient was further investigated. The organ coefficient of adipate in DIO mice significantly increased, while the organ coefficient of liver decreased compared with the control mice. The changes in the liver may not be consistent with our expectations. As obese mice showed excessive fat deposition, the organ coefficient of adipose tissue increased. And the weight gain of the liver is less than the overall weight gain of mice, so the organ coefficient of the liver descended. NITyr improved the above phenomenon. The above results indicated that NITyr did interfere with fat synthesis or decomposition, while its effect on the liver is unclear. Therefore, we further discussed the pathological sections and indexes of fat and liver.

Adipocyte enlargement and hepatic steatosis are essential features of obesity (29). NITyr reduced the area of individual adipocytes and increased the number of adipocytes, consistent with the above results. As we know that the liver is the most important metabolic organ responsible for lipid metabolism including lipogenesis, lipolysis, and lipid oxidation, which maintains lipid homeostasis (30, 31). Thus, the liver is the hub of fat transport. The liver is one of the organs that severely suffer from obesity. Obesity disrupted the overall metabolic function of the liver to promote the accumulation of fat in the liver to form “fatty liver”, which further aggravated obesity, resulting in a vicious circle (32, 33). Thus, if there were methods to intervene in hepatic lipid metabolism, they would be expected to offer a potential strategy for obesity alleviation. Due to the extremely high sensitivity of ALT and AST (34), they are used as indicators to evaluate liver injury clinically. TNF-α and IL-1β reflected the inflammatory status of the liver (35). Compared with the control group, a notable increase in ALT, AST, TNF-α and IL-1β was detected in the DIO mice. NITyr reduced the ALT and AST but didn’t affect the level of TNF-α and IL-1β. The liver of DIO mice didn’t show steatosis or inflammatory infiltration, and no significance was observed after NITyr intervention. The above results showed that the liver injury of DIO mice established by our research group was not noticeable.

DIO is often accompanied by abnormal metabolism of glucose and lipids in the body (36), so TG, TC, glucose and insulin levels in serum were detected. NITyr reduced the high levels of TG, TC and glucose induced by DIO mice, indicating that the glucose and lipid metabolism in DIO mice was unbalanced, and NITyr intervention restored the glucose and lipid balance. Due to insulin deficiency or resistance caused by hyperglycemia (37), the insulin levels were tested. Insulin levels in the DIO group were significantly increased compared to that in the control group. In contrast, neither group under drug treatment showed significant differences, indicating the effect of NITyr on regulating blood glucose is independent of insulin.

Ghrelin produced by P/D1 cells at the bottom of the stomach promotes appetite (38). PYY, a derived intestinal hormone, makes the body feel full, and then reduces food intake (39). In the study, the PYY and ghrelin levels of DIO mice increased compared with the control group. The food intake and water consumption of DIO mice decreased compared with the control group. On the one hand, the increase of PYY levels induced by HFD is higher than that of ghrelin; on the other hand, mice fed a high-fat diet rich in energy for a long time will produce a strong sense of satiety, thus long-term consumption of high-fat diet inhibited the appetite of mice. NITyr didn’t affect PYY and ghrelin levels, suggesting that the weight loss induced by NITyr was independent of food intake. In the previous studies, NITyr as a neuroprotective agent enhanced BDNF levels in the brain of mice (data unpublished), and BDNF regulated food intake and energy metabolism (40). Thus, the levels of BDNF were investigated in our study. NITyr didn’t interfere with the BDNF level in DIO mice, further indicating that the weight loss induced by NITyr was independent of appetite.

PPAR, a transcriptional regulator of adipogenesis, contributes to lipid accumulation and adipocyte differentiation. It was found to be activated in the process of adipogenesis and regulate the expression of AMPK (41). AMPK is an AMP-dependent protein kinase, which plays a role in energy homeostasis through the upregulation of catabolic processes that generate ATP. AMPK activation enhances the catabolism of the body, and reduce the expression of lipid synthesis-related factors such as FAS to regulate the synthesis and utilization of lipids (42). FAS is a key enzyme in lipogenesis, and when activated, it increases fatty acid synthesis and insulin resistance in adipose tissues. In the present study, we found that PPAR and FAS were up-regulated in HFD mice, the expression of which was down-regulated by the introduction of NITyr. P-AMPK was lowly expressed in adipose tissues in HFD mice, while highly expressed after NITyr administration.

The endocannabinoid system consists of endocannabinoid (AEA), cannabinoid receptors (CB_1_ and CB_2_) and hydrolase (FAAH). The inhibition of FAAH increased the AEA level, thus indirectly activating the CB_1_ and CB_2_ receptors (43). Activation of the CB_1_ receptor promoted weight gain, while activation of the CB_2_ receptor urged weight loss (13). In the study, NITyr upregulated CB_2_ expressions and downregulated CB_1_ expressions. We speculated that the downregulation of the CB_1_ receptor was due to receptor desensitization caused by long-term drug action. Besides, CB_2_ receptors were more stable; thus, they were only up-regulated even after long-term drug stimulation. In addition, OEA and PEA, as AEA analogs, activated TRPV1 and PPAR receptors to promote weight loss. Hence, NITyr as an AEA analog, may also bring a similar role to OEA and PEA (44).

Overall, NITyr fight against obesity *via* balance glycolipid metabolism involved in CB_1_ and CB_2_ activation (**Figure 8**).

## Data availability statement

The original contributions presented in the study are included in the article/**Supplementary Materials**. Further inquiries can be directed to the corresponding author.

## Ethics statement

The animal study was reviewed and approved by Animal Experiment Ethics Committee of Chengdu Medical College.

## Author contributions

Methodology, Z-YY and Y-YW. Project administration, Z-YY and J-HZ. Data curation and analysis, YZ and Y-QY. Writing-original draft manuscript, SL and TH. Writing-review, SL. Funding acquisition, SL and TH. All authors approved the published version of the manuscript.

## Funding

This research was funded by the National Natural Science Foundation of China (grant number 81803514), the Foundation of Science and Technology Department of Sichuan Province (grant number 22NSFSC0727), Disciplinary Construction Innovation Team Foundation of Chengdu Medical College (grant number CMC-XK-2104),Scientific research project of Sichuan Medical Association (grant number S19078), Chengdu Municipal Health Commission (grant number 2020163).

## Conflict of interest

YZ was employed by Sichuan Yuanda Shuyang Pharmaceutical Co.

The authors declare that the research was conducted in the absence of any commercial or financial relationships that could be construed as a potential conflict of interest.

## Publisher’s note

All claims expressed in this article are solely those of the authors and do not necessarily represent those of their affiliated organizations, or those of the publisher, the editors and the reviewers. Any product that may be evaluated in this article, or claim that may be made by its manufacturer, is not guaranteed or endorsed by the publisher.
